# An Analytic Model for Reducing Authentication Signaling Traffic in an End-to-End Authentication Scheme

**DOI:** 10.3390/s21154980

**Published:** 2021-07-22

**Authors:** Shadi Nashwan, Imad I. H. Nashwan

**Affiliations:** 1 Computer Science Department, Jouf University, Sakaka 42421, Saudi Arabia; 2 Faculty of Technology and Applied Science, Al Quds Open University, Gaza 860, Palestine; inashwan@qou.edu

**Keywords:** E2EA scheme, healthcare IoT system, WMSN, mutual authentication, Poisson process, probability distribution

## Abstract

In an end-to-end authentication (E2EA) scheme, the physician, patient, and sensor nodes authenticate each other through the healthcare service provider in three phases: the long-term authentication phase (LAP), short-term authentication phase (SAP), and sensor authentication phase (WAP). Once the LAP is executed between all communication nodes, the SAP is executed (*m*) times between the physician and patient by deriving a new key from the PS*ij* key generated by healthcare service provider during the LAP. In addition, the WAP is executed between the connected sensor and patient (*m* + 1) times without going back to the service provider. Thus, it is critical to determine an appropriate (*m*) value to maintain a specific security level and to minimize the cost of E2EA. Therefore, we proposed an analytic model in which the authentication signaling traffic is represented by a Poisson process to derive an authentication signaling traffic cost function for the (*m*) value. wherein the residence time of authentication has three distributions: gamma, hypo-exponential, and exponential. Finally, using the numerical analysis of the derived cost function, an optimal value (*m*) that minimizes the authentication signaling traffic cost of the E2EA scheme was determined.

## 1. Introduction

Today, the Internet of Things (IoT) healthcare system is in common use around the world. Its essential goal is to monitor a patient’s vital signs while a physician delivers treatment and medical advice remotely; moreover, it can reduce the number of the healthcare centers and bring expert medical care to remote areas where there is a shortage of them [1,2,3,4,5,6].

A wireless medical sensor network (WMSN) collects data from sensors that register temperature, blood pressure, blood sugar levels, etc. [1,2,3,4,5]. Then, the data are transmitted to the healthcare provider, which sends them to physicians electronically [1,2,7]. In such a system, data security is the main concern because an unauthorized party could access a patient’s sensor nodes to reveal the secrecy and privacy of his or her health status [1,2,8]. Furthermore, the unauthorized party could compromise the integrity of the patient safety by falsifying the doctor’s instructions or advice or by changing a dose from the electronic insulin pumps [1]. Therefore, the healthcare IoT system is susceptible to numerous types of attacks such as smartcard loss, sensor spoofing, desynchronization, impersonation, replay, insider, intrusion, and man-in-the-middle attacks [1,2,9,10,11].

Several authentication schemes have been proposed to deal with sensor deficiencies, but they did not adequately consider performance and authentication costs [12,13,14,15,16,17,18,19,20,21,22,23,24,25]. To reduce authentication overhead, communication has been made more practical. Many schemes now generate a preset number of parameters to execute more authentication sessions between system nodes without having to refer back to the authentication center or the service provider’s server, thus reducing delays. However, this technique could have adverse results if some of the authentication parameters have to be changed because of, for example, a difference in the request rate. Therefore, authentication schemes need to use a cost function that estimates the number of the authentication sessions and the quantity of authentication parameters to be generated.

The first author has proposed an authentication scheme called end-to-end authentication (E2EA) [1], which can support various security and performance features such that mutual authentication, anonymity, and perfect forwarding services are satisfied. Furthermore, E2EA can protect against the abovementioned attacks using low-cost storage space, computations, and communications.

Therefore, in this paper we proposed an analytical cost function model to examine the effect of the number of authentication parameters that will be generated during the execution of E2EA on the signaling traffic cost. Thus, the healthcare service provider can estimate in advance the number authentication sessions to be executed for a specific patient; then, according to this cost estimate, set the number of parameters to be generated and transmit them to the nodes when the E2EA scheme is executed.

### 1.1. Background

**Figure 1:**
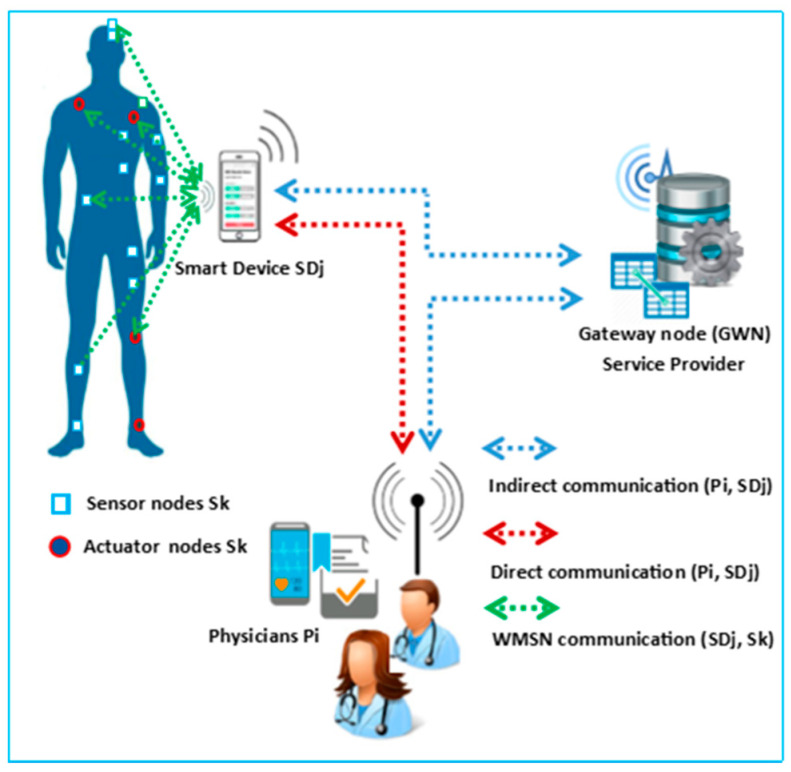
Healthcare IoT system architecture of E2EA.

In E2EA, the communication nodes of the IoT architecture are the gateway node (GWN), representing the healthcare service provider, the physician’s monitoring device (P*i*), the patient’s smart device (SD*j*), and the nodes (S*k*) as illustrated in Figure 1. The S*k* sensor nodes collect the patient’s vital signs and send them as an on-demand report to the SD*j*; the S*k* actuator nodes receive medical orders from the P*i* through the SD*j* to perform a specific action such as turning on the insulin pumps [1,2,3,4,5,6]. Communication between the SD*j* and S*k* nodes is accomplished via the WMSN [1,2,3,4,5,6,12].

The SD*j* supports the registration process with the GWN and connects with a new sensor node. The SD*j* should be able to save the vital signs collected by specific sensor node, then forward them to the P*i* indirectly through GWN or directly during emergencies. Communication between the SD*j*, GWN, and P*i* is conducted over the Internet [1,12,13,14,15,16].

The GWN is the core node of the E2EA scheme because it supports registration with the P*i* and SD*j*. The GWN observes the authentication and key agreement (AKA) execution to coordinate authentication between the P*i* and SD*j*.

The P*i* can collect vital signs from the SD*j* and transmit medical orders to the actuator sensors for treatment through the SD*j*.

**Figure 2:**
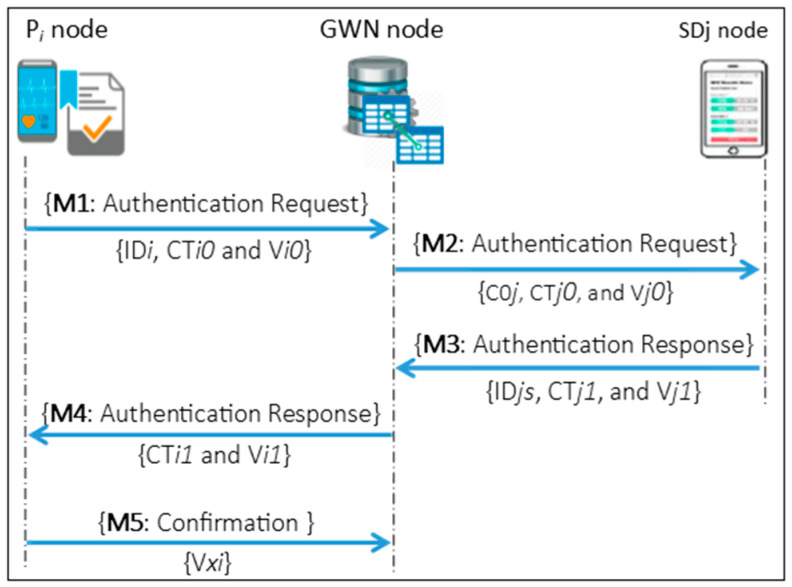
Long-term authentication phase (LAP).

**Figure 3:**
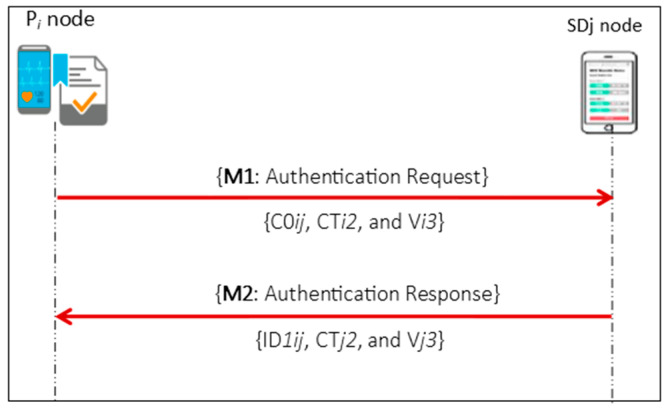
Short-term authentication phase (SAP).

**Figure 4:**
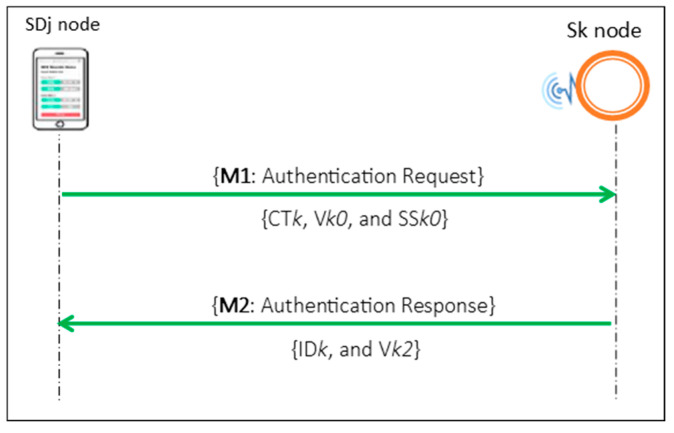
WMSN authentication phase (WAP).

In E2EA, authentication is exercised for every monitoring and treatment event between the GWN, P*i*, SD*j* and S*k* through three authentications phases: the long-term authentication phase (LAP), short-term authentication phase (SAP), and WMSN authentication phase (WAP) as shown in Figure 2, Figure 3 and Figure 4, respectively.

As shown in Figure 2, the LAP supports full mutual authentication, i.e., authentication of the P*i* by the GWN and authentication of the GWN by the P*i* through the exchange of authentication messages M1, M4 and M5. Furthermore, authentication of the GWN by the SD*j* and authentication of the SD*j* by the GWN through exchanging the authentication messages M2 and M3.

The LAP performs a set of a symmetric cryptographic functions using the authentication keys that were generated during the registration phases of the P*i* and SD*j* with the GWN. Besides, one-way hash functions are used to generate the verification values of the authentication parameters for all authentication messages. This phase also establishes a new subsequent key PS*ij* generated by the GWN to be used when the P*i* and SD*j* execute the SAP to authenticate each other directly.

M1 is a request authentication message that the P*i* generates to prove itself to the GWN and has the values ID*i*, CT*i0* and V*i0*: ID*i* represents the P*i*’s identity; CT*i0* is an encrypted value of the P*i*’s timestamp and a random number with the identity of the patient; and V*i0* is a hash value used on the GWN side to verify the CT*i0* value. M4 is a response message that the GWN generates to prove itself to the P*i* and has the values CT*i1* and V*i1*: CT*i1* is an encryption of the concatenation value of the timestamp, random number, and PS*ij* key that are generated by the GWN, and V*i1* is a hash value used on the P*i* side to verify the CT*i1* value. M5 is a confirmation message the P*i* sends to the GWN to complete the mutual authentication. This message includes the hash value (V*xi*), which is used as a confirmation value to the GWN.

On the other side, M2 is a request authentication message that the GWN generates to prove itself to the SD*j* and has the values C0*j*, CT*j0*, and V*j0*: C0*j* is an incremental counter of the authentication session; CT*j0* is an encrypted value of the timestamp, random number, the PS*ij* key of the GWN’s; and V*j0* is a hash value used on the SD*j* side to verify the CT*j0* value. Finally, M3 is a response message that the SD*j* generates to prove itself to the GWN and has the values ID*js*, CT*j1*, and V*j1*: ID*js* is the SD*j*’s identity; CT*j1* is an encrypted value of the SD*j*’s timestamp and random number; and V*j1* is a hash value used on the GWN side to verify the CT*j1* value.

In the SAP, as illustrated in Figure 3, mutual authentication is achieved between the P*i* and SD*j* through the direct exchange of authentication messages M1 and M2. The PS*ij* that was received by both sides during the LAP will be used to encrypt the authentication parameters. In this phase, both authentication sides maintained a session counter (C0*ij*) to determine how many times the PS*ij* value will derive a new key for the next direct mutual authentication session without going back to execute the LAP for a new PS*ij* key. M1 is a request authentication message generated by the P*i* to prove itself to the SD*j* and has the values C0*ij*, CT*i2*, and V*i3*: C0*ij* is a session counter as mentioned; CT*j2* is an encrypted value of the P*i*’s timestamp and random number with C0*ij* using the derived subsequent key (PS*ij*); and V*i3* is a hash value used on the SD*j* side to verify the CT*i2* value. On other hand, the M2 message is a response message that the SD*j* generates to prove itself to the P*i*. In the same manner, M2 comprises ID*1ij*, CT*j2*, and V*j3*: ID*1ij* represents the pseudonym for SD*j* generated by the P*i* to derive a new value of the PS*ij* key for the current authentication session, and V*j3* is a hash value on the P*i* side that verifies the CT*j2* value.

As shown in Figure 4, the exchange of M1 and M2 achieves mutual authentication between the SD*j* and S*k* in the WAP. The SD*j* generates a secret key (SK*k*) to calculate the authentication parameters of the request message by performing a set of one-way hash functions, and the S*k* derives the same SK*k* value to calculate the authentication parameters of the response message using the same hash functions that used on the SD*j* side. In this phase, both of the authentication sides maintain a pair of sequence numbers, SS*k0* and SS*k1*, to maintain mutual synchronization.

M1 is a request authentication message that is the SD*j* generates to prove itself to the connected S*k* and has the values CT*k*, V*k0* and SS*k0*: CT*k* hides the hash value of the SK*k* and the authentication session number; V*k0* is a hash value on the S*k* side that verifies CT*k*; and SS*k0* is a sequence number on the SD*j* side. Finally, M2 is a response massage that the S*k* generates to prove itself to the SD*j* and consists of ID*k* and V*k2:* ID*k* is a pseudonym for the S*k* generated by the SD*j* to identify the S*k*, and V*k2* value is a hash value used on the SD*j* side to verify the connected S*k*.

From the aforementioned discussion, the main execution points of the E2EA scheme can be summarized as follows:

(1) The P*i* executes the LAP by sending an authentication request message to the GWN and delegates the GWN to perform mutual authentication with the SD*j*, wherein both of the P*i* and SD*j* obtain the seed value of the PS*ij* key;
(2) The P*i* and SD*j* can execute the SAP to authenticate each other a maximum of *m* times directly without going back to execute the LAP. In each SAP execution, the P*i* and SD*j* derive a new value from the PS*ij* key to encrypt the authentication parameters of the messages exchanged between them;
(3) The WAP can be executed between the SD*j* and connected S*k* after either the LAP or SAP execution to exchange either the vital signs or the medical orders of the patient. Therefore, the WAP can execute a maximum of *m* + 1 times without going back to the LAP execution.

**Figure 5:**
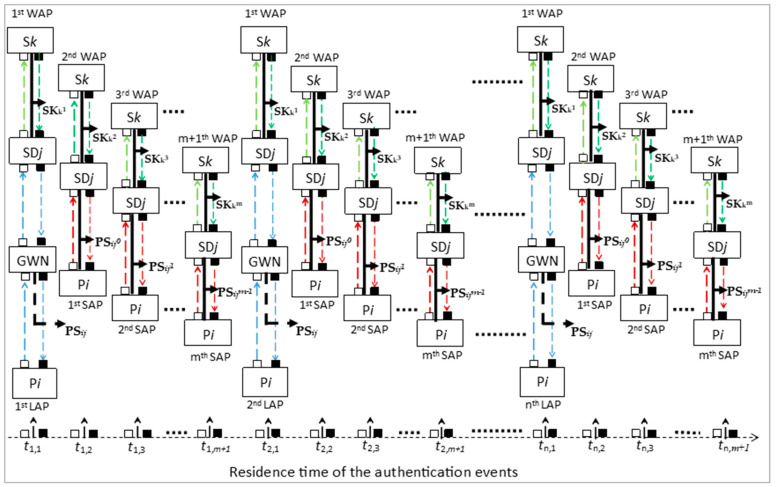
The E2EA scheme residence timeline diagram: the dashed blue arrows represent the request and response authentication messages of the LAP; the dashed red arrows represent the request and response authentication messages of the SAP; the dashed green arrows represent the request and response authentication messages of the LAP; the dashed black arrows represent the generation process of the PS*ij* key; and the solid black arrows represent the derivate process of subsequent PS*ij* and SK*k* keys.

For further clarification of the relationship among the three phases, consider the timeline diagram in Figure 5. Suppose that the P*i* sends a new authentication request to the GWN at time **τ**_1,1_. Then, the LAP is executed and a new PS*ij* key is created by the GWN. So, both of the P*i* and SD*j* obtained the first value of the PS*ij*^0^ key. Mutual authentication is performed between the SD*j* and S*k* by executing WAP using the first value of SK*k*^1^.

After **τ**_1,1_, the second authentication request event occurs at time **τ**_1,2_. The P*i* initiates the first SAP using the (PS*ij*^0^) key and the SD*j* initiates the second WAP with S*k* using the second derived value of (SK*k*^2^).

At time **τ**_1,*m*+1_, the last allowable derived key value (PS*ij^m^*^−1^) for the PS*ij* key was used for the SAP at the *m*-th authentication event. (C*ij* is at the maximum value of *m* − 1). Moreover, based on the new value of SS*k0* and SS*k1*, the last allowable derived value of SK*k*^m^ was used for WAP at the (*m*+1)-th authentication event. So, at time **τ**_1,*m*+1_, both the P*i* and SD*j* used a set of derived subsequent keys {PS*ij*^0^, PS*ij*^1^, PS*ij*^2^…., PS*ij^m^*^−1^} to authenticate each other by executing *m*-SAPs directly.

After **τ**_1,*m*+1_, the next authentication event occurred at **τ**_2,1_. The P*i* realized that the value of C*ij* had reached maximum (C*ij* = *m* − 1), which executed the second LAP to obtain the next PS*ij* key from the GWN, after which P*i* and SD*j* performed the *m*-SAPs and *m*+1-WAPs, respectively. For next authentication events, the LAPs, SAPs, and WAPs were performed accordingly as descried above.

After **τ***_n_*_,*m*+1_, the P*i* and SD*j* used the *N*-th PS*ij* values that was created by GWN via all executed LAPs. It is worth mentioning that, the first WAP execution in each of the LAPs were not considered since it was not included in min C*ij*–max C*ij*. Thus, during the period **τ**_1,1_–**τ***_n_*_,*m*+1_, the authentication sessions number is (*N* − 1 LAPs, (*N* − 1) × *m* SAPs and (*N* − 1) × *m* WAPs).

### 1.2. Related Work

A few researchers have proposed an analytical model for the traffic signaling of authentication schemes. In 2003, Lin and Chen [26] proposed an analytical model base on the Poisson process to reduce authentication signaling traffic in a third-generation mobile network. This model was proposed to investigate the impact of the number of authentication vectors (AVs) generated by the serving network on the signaling traffic during the execution of the authentication scheme. This model was also used to develop an automatic K-selection mechanism that selected the size of the AV array dynamically to reduce network signaling cost. In 2009, Hen et al. [27] evaluated the signaling loads in the third-generation mobile network via an analytical model based on the renewal process theory. This model was used to study the effect of the call arrival rate, mobility, subscribers’ preference and operational policy during execution of the scheme. In 2017, Al-Saraireh [28] proposed an analytic model based on the Poisson process to reduce authentication signaling traffic in the long term evolution (LTE) mobile network. This model was proposed to determine the impact of the size of authentication vector (AV) array generated by the serving network on the signaling traffic during the execution. In 2021, the authors [29] proposed an analytical model to reduce the overhead message cost of the secure anonymity authentication key and key agreement scheme (SAK–AKA) for 4G/5G mobile networks. In this analytical model, the authentication messages were represented by a Poisson process, wherein the residence time of the user request for authentication had an exponential distribution to determine the number of authentication vectors (AVs) to be generated by the serving network to authenticate the user’s mobile.

In none of the aforementioned research papers was there a proposal for an analytical model to analyze and minimize the authentication signaling traffic cost of a healthcare systems authentication scheme.

### 1.3. Motivations and Contributions

In an E2EA scheme, LAP operations carry high communication costs. Therefore, we sought to increase the maximum limit of C*ij* to reduce the number of LAPs performed when the P*i* sends an authentication request to the GWN. On the other hand, if there is a large number of *m*, the level of security may be degraded. Thus, an appropriate (*m*) value need to be found that can maintain a specific level of security while minimizing the authentication signaling traffic costs. The main contributions of this paper can be summarized as follows:

(1) Introduced the E2EA scheme by explaining the relationship between its authentication phases.
(2) Introduced the residence timeline of authentication events in E2EA scheme.
(3) Proposed an analytic model to represent E2EA signaling traffic according to Poisson process, wherein the residence authentication time has three types of distribution: gamma, hypo-exponential, and exponential.
(4) Derived a signaling traffic cost function for the (*m*) value effect on the communication lines between the authentication nodes.
(5) Analyzed the derived signaling traffic cost function numerically using the Newton–Raphson method to determine the optimal value of (*m*) to minimize the cost of E2EA scheme.

### 1.4. Organization of This Paper

In Section 2, an analytic model is proposed to derive an authentication signaling traffic cost function for the E2EA scheme by representing the signaling traffic according to the Poisson process using three types of distributions. Section 3 discusses the analysis of the proposed analytical model to show the impact of the (*m*) value on the signaling traffic costs of the authentication events. In Section 4, the Newton–Raphson method is used to derive the optimal value of (*m*) numerically. Finally, we provide our conclusions in Section 5.

## 2. Proposed Analytic Model of E2EAScheme

Let *N* be the total number of LAP authentication events performed by the P*i*. For each LAP event, the P*i* and SD*j* execute *m*-SAPs, where the WAPs are a consequence of the SAP times. Suppose that the aggregate incoming/outgoing P*i* authentication messages form a Poisson process with rate (*λ*)*,* {N(*t*): t ≥ 0}, where *t* is the residence time that the P*i* sends an authentication request to the GWN. Let Ψ (*n*, *m*, *t*) be the probability that there are *n*-LAPs for residence period *t;* this means that the process does not reach the (*n*+1)-th LAP and the authentications were *n*-LAPs; that is, *m*(*n* − 1)-SAPs and *i*-SAPs before time **τ** *_n_*_,*m*+1_, where 0 ≤ *i* ≤ *m* − 1. Thus, the total number of performed authentication events of the P*i* at time *t* = (**τ***_n_*_,*m*+1_–**τ**_1,1_) is (*m*(*n* − 1) *+ i*). Therefore, according the probability function of the Poisson distribution [30], we have:

(1) $$\Psi \left(n,m,t\right)=\sum_{i=0}^{m-1}\frac{{\left(\lambda t\right)}^{\left(n-1\right)m+i}}{\left[\left(n-1\right)m+i\right]!}e^{-\lambda t}$$

let Ψ (*n*, *m*) be the probability function that there are *n*-LAPs during the residence time and *m* is the performed SAPs for each LAP so that:

(2) $$\Psi \left(n,m\right)=\int_{0}^{\infty }P\left\{N=n|T=t\right\}f\left(t\right)dt=\int_{0}^{\infty }\Psi \left(n,m,t\right)f\left(t\right)dt$$

where *T* is a non-negative random variable representing the residence time of the P*i*. The expected number of authentication events through the residence time is given as:

(3) $$E\left(N\right)=\sum_{n=1}^{\infty }n\times \Psi \left(n,m\right)$$

if *C*(*m*) is considered to be the total cost of transmitted messages in the E2EA scheme through the residence time when the P*i* requests authentication to monitor a specific SD*_j_*, then the total cost of all authentication phases is the expected number of authentication events multiplied by the cost of each event (i.e., the LAPs, SAPs, and WAPs phases), which can be expressed as:

(4) C(m)=E(N)×[5α+2(α+β)m]

where *α* and *β* represent the overhead transmission cost of the authentication messages through the internet and WMSN connections. In the following subsections, the Ψ (*n*, *m*), *E*(*N*), and *C*(*m*) are computed, wherein the residence time *T* has gamma, hypo-exponential, and exponential distributions, respectively.

### 2.1. T Has an Exponential Distribution with Mean $\mu^{-1}$

Equation (2) becomes:

$$\Psi \left(n,m\right)=\sum_{i=0}^{m-1}\int_{0}^{\infty }\mu \frac{\lambda^{\left(n-1\right)m+i}}{\left[\left(n-1\right)m+i\right]!}e^{-\left(\lambda +\mu \right)t}dt=\sum_{i=0}^{m-1}\left(\frac{\mu }{\lambda +\mu }\right){\left(\frac{\lambda }{\lambda +\mu }\right)}^{\left(n-1\right)m+i}$$

Using the geometric series formula:

(5) $$\Psi \left(n,m\right)={\left(\frac{\lambda }{\lambda +\mu }\right)}^{\left(n-1\right)m}\left[1-{\left(\frac{\lambda }{\lambda +\mu }\right)}^{m}\right]$$

if $\gamma =\frac{\lambda }{\lambda +\mu }$, and $p=1-\gamma^{m}$; then Equation (5) becomes:

(6) $$\Psi \left(n,m\right)=p{\left(1-p\right)}^{n-1}\;\;n=1,2,\ldots$$

Equation (6) explains that Ψ (*n*, *m*) has the geometric probability function with mean *p*^−1^. This is a reasonable and consistent result since a LAP should be executed first and then *m*-SAPs with probability *γ^m^*. In general, *N* has a geometric distribution expectation, so (3) and (4) can be rewritten as (7) and (8), respectively:

(7) $$E\left(N\right)=\sum_{n=1}^{\infty }n\times \Psi \left(n,m\right)=\frac{1}{p}=\frac{1}{1-\gamma^{m}}$$

(8) $$C\left(m\right)=\frac{5\alpha +2\left(\alpha +\beta \right)m}{1-\gamma^{m}}$$

### 2.2. T Has Hypo-Exponential Distribution

Actually, the hypo-exponential distribution was used for modeling multiple exponential phases in series, which is a suitable for an IoT system since the P*i* executes two types of authentication phases (LAP and SAP). WLOG, assume that *T* has hypo-exponential distribution with mean $\mu_{1}^{-1}+\mu_{2}^{-1}$ such that $\mu_{1}\neq \mu_{2}$, then from Equation (2) we have:

$$\Psi \left(n,m\right)=\sum_{i=0}^{m-1}\int_{0}^{\infty }\frac{\lambda^{\left(n-1\right)m+i}}{\left[\left(n-1\right)m+i\right]!}e^{-\lambda t}\frac{\mu_{1}\mu_{2}}{\mu_{2}-\mu_{1}}\left(e^{-\mu_{1}t}-e^{-\mu_{2}t}\right)dt$$

$$\Psi \left(n,m\right)=\sum_{i=0}^{m-1}\left[\left(\frac{\mu_{2}}{\mu_{2}-\mu_{1}}\right)\left(1-\gamma_{1}\right)\gamma_{1}{}^{\left(n-1\right)m+i}-\left(\frac{\mu_{1}}{\mu_{2}-\mu_{1}}\right)\left(1-\gamma_{2}\right)\gamma_{2}{}^{\left(n-1\right)m+i}\right]$$

If $p_{j}=1-\gamma_{j}^{m},j=1,2$, then the geometric series formula gives:

(9) $$\Psi \left(n,m\right)=\left(\frac{\mu_{2}}{\mu_{2}-\mu_{1}}\right)p_{1}{}^{\left(n-1\right)}\left[1-p_{1}\right]-\left(\frac{\mu_{1}}{\mu_{2}-\mu_{1}}\right)p_{2}{}^{\left(n-1\right)}\left[1-p_{2}\right]:n=1,2,\ldots$$

Note that the Ψ(n,m) is a linear combination of two probability density functions of the geometric distribution with means $\frac{1}{p_{1}}$ and $\frac{1}{p_{2}}$, respectively; therefore:

(10) $$E\left(N\right)=\frac{\mu_{2}p_{2}-\mu_{1}p_{1}}{\left(\mu_{2}-\mu_{1}\right)p_{1}p_{2}}=\frac{\mu_{2}\left(1-\gamma_{2}^{m}\right)-\mu_{1}\left(1-\gamma_{1}^{m}\right)}{\left(\mu_{2}-\mu_{1}\right)\left(1-\gamma_{1}^{m}\right)\left(1-\gamma_{2}^{m}\right)}$$

(11) $$C\left(m\right)=\frac{\left[\mu_{2}p_{2}-\mu_{1}p_{1}\right]\left[5\alpha +2(\alpha +\beta )m\right]}{\left(\mu_{2}-\mu_{1}\right)p_{1}p_{2}}$$

### 2.3. T Has a Gamma Distribution

Assuming that *T* has a gamma distribution with the shape parameter κ>0 and that θ is the scale parameter (with mean $\mu^{-1}$, and variance ν), then from Equation (2) we have:

(12) $$\Psi \left(n,m\right)=\int_{0}^{\infty }\sum_{i=0}^{m-1}\frac{{\left(\lambda t\right)}^{\left(n-1\right)m+i}}{\left(\left(n-1\right)m+i\right)!}e^{-\lambda t}\frac{\theta^{\kappa }t^{\kappa -1}e^{-\theta t}}{\Gamma \left(\kappa \right)}dt=\sum_{i=0}^{m-1}\frac{\Gamma \left[\left(n-1\right)m+i+\kappa +1\right]}{\Gamma \left(\left(n-1\right)m+i\right)\Gamma \left(\kappa \right)}{\left(1-\gamma \right)}^{\left(n-1\right)m+i}\gamma^{\kappa }$$

where $\gamma =\frac{\theta }{\lambda +\theta }$.

Ψ (*n*, *m*) is the cumulative distribution function of the negative binomial distribution regarding the number of executed *m*-SAPS (sometimes called mixture of a family of Poisson distributions with Gamma mixing weights) with parameter (κ) and (*γ*). To find the relation between the probability function Ψ (*n*, *m*) and the mean of the residence time, substitute $\kappa \theta^{-1}=\mu^{-1}$ and $\nu =\kappa \theta^{-2}$ into Equation (12):

(13) $$\Psi \left(n,m\right)=\sum_{i=0}^{m-1}\frac{{\left(\lambda \mu \nu \right)}^{\left(n-1\right)m+i}}{\left[\left(n-1\right)m+i\right]!}\left(\prod_{j=1}^{\left(n-1\right)m+i}\left[{\left(\mu^{2}\nu \right)}^{-1}+1\right]\right){\left(\lambda \mu \nu +1\right)}^{-\left[{\left(\mu^{2}\nu \right)}^{-1}+\left(n-1\right)m+i\right]}$$

Thus, the expectation *E*(*N*) and the cost function *C*(*m*) in Equations (3) and (4) will be:

(14) $$E\left(N\right)=\sum_{n=1}^{\infty }n\times \left(\sum_{i=0}^{m-1}\frac{{\left(\lambda \mu \nu \right)}^{\left(n-1\right)m+i}}{\left[\left(n-1\right)m+i\right]!}\left(\prod_{j=1}^{\left(n-1\right)m+i}\left({\left(\mu^{2}\nu \right)}^{-1}+1\right)\right){\left(\lambda \mu \nu +1\right)}^{-\left[{\left(\mu^{2}\nu \right)}^{-1}+\left(n-1\right)m+i\right]}\right)$$

(15) $$\begin{array}{l} C\left(m\right)=\left[5\alpha +2\left(\alpha +\beta \right)m\right]\times \\ \;\;\;\;\;\left[\sum_{n=1}^{\infty }n\times \left(\sum_{i=0}^{m-1}\frac{{\left(\lambda \mu \nu \right)}^{\left(n-1\right)m+i}{\left(\lambda \mu \nu +1\right)}^{-\left[{\left(\mu^{2}\nu \right)}^{-1}+\left(n-1\right)m+i\right]}}{\left[\left(n-1\right)m+i\right]!}\prod_{j=1}^{\left(n-1\right)m+i}\left({\left(\mu^{2}\nu \right)}^{-1}+1\right)\right)\right] \end{array}$$

## 3. Analysis of the Proposed Analytical Model

This section describes the impact of (*m*) values on the *E*(*N*) according to Equations (7), (10) and (14), and the cost function *C*(*m*) according to Equations (8), (11) and (15).

**Figure 6:**
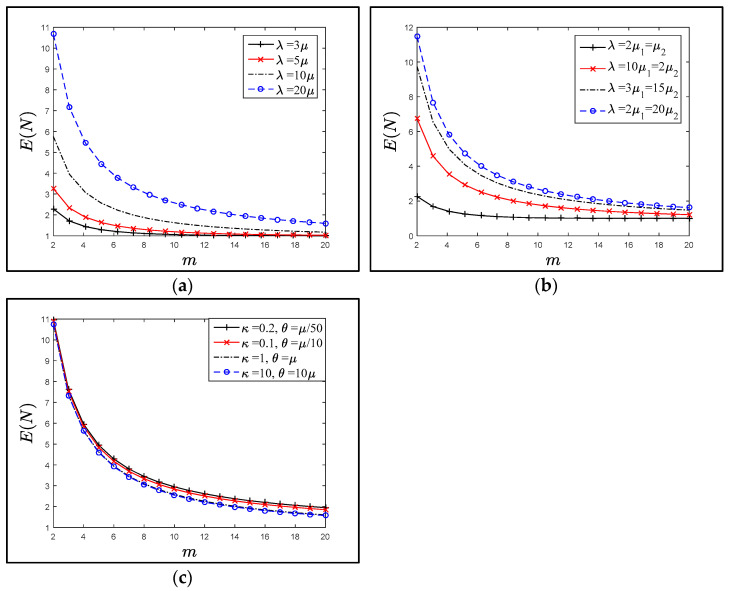
Effect of SAPs on the expected LAPs when the residence time is distributed as in (**a**–**c**). (**a**) Exponentially distributed residence time with mean $\mu^{-1}$. (**b**) Hypo exponential distributed residence time with mean $\mu_{1}^{-1}+\mu_{2}^{-1}$. (**c**) Gamma distributed residence time, when λ=20μ.

Figure 6a–c plot the relation between the *E*(*N*) versus the value of *m* for the multiple arrival rate (*λ*), where the residence time is distributed (exponential, hypo exponential and gamma) with means *μ*^−1^, *μ*_1_^−1^ + *μ*_2_^−1,^ and *μ*^−1^, respectively. It is obvious the *E*(*N*) is a decreasing function of *m* and the plotted points are closed to each other. After a while *m* ≥ 10, *E*(*N*) is insignificantly reduced by increasing the value of *m*.

**Figure 7:**
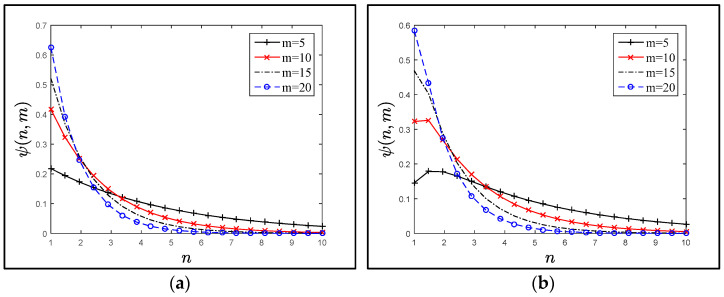

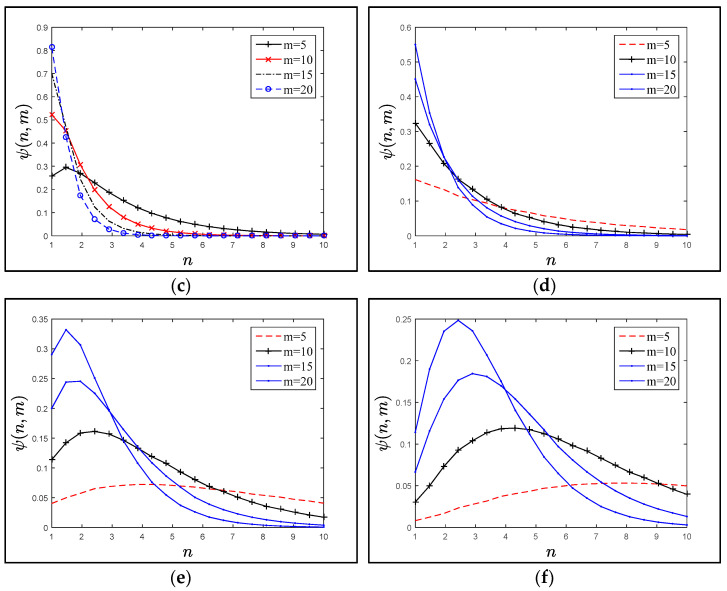
With different residence/request time distributions as in (**a**–**e**). (**a**) Exponentially distributed residence time, mean $\mu^{-1}$, when λ=10μ. (**b**) Hypo-exponential distributed residence time, when $\lambda =2\mu_{1}=20\mu_{2}$. (**c**) Hypo-exponential distributed residence time, when $\lambda =10\mu_{1}=2\mu_{2}$. (**d**) Gamma-distributed residence time is when κ=1, and λ=10μ. (**e**) Gamma-distributed residence time, when κ=2, and λ=20μ. (**f**) Gamma-distributed residence time, when κ=3, and λ=30μ.

On the other hand, the function Ψ (*n*, *m*) had a different behavior with respect to *m*, for the fixed ratio *γ*. Figure 7a–f plot the probability density function Ψ (*n*, *m*) when the number of SAPs was 5 ≤ *m* ≤ 20, for various residence-time distributions. Notice that the behavior of Ψ (*n*, *m*) was similar after a specified number of *n*; for *n* ≥ 6, the plotted points were closed to each other. This observation was consistent with Figure 6, i.e., the *E*(*N*) value was the same for the large (*m*) value, and the increasing value of *m* did not improve the *E*(*N*) value.

**Figure 8:**
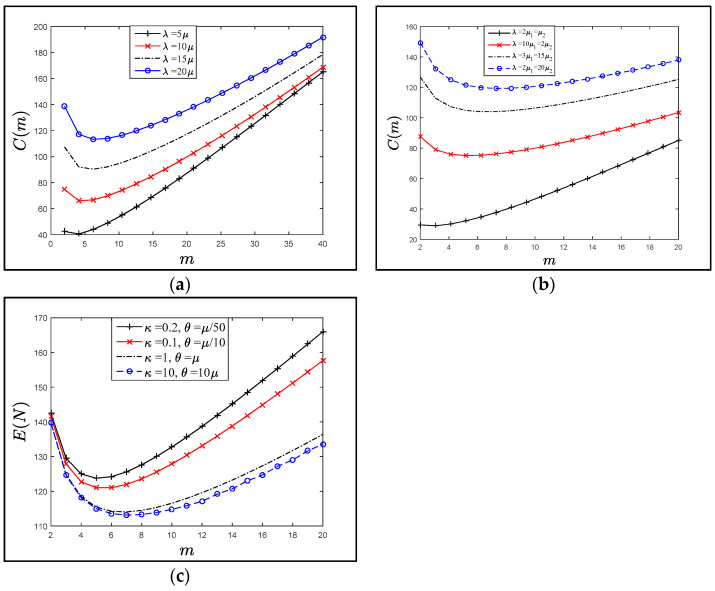
The cost function C(m) when α=β=1, with different residence time distributions as in (**a**–**c**). (**a**) The residence time is exponential distributed. (**b**) The residence time is hypo exponential distributed. (**c**) The residence time is gamma distributed.

Figure 8a–c show the effect of *m* values on the trend of the cost function C(*m*) for fixed α, β, and *λ*. The trend of the plots is the same for various residence time distribution, all plots obviously show that there is a critical value (*m*), which is minimizing the cost function, and after this point, the C(*m*) is rapidly increased. Also, the C(*m*) values are significantly increased with the increasing of the (*λ*) values. These results are proportionate with goal of the direct authentication between the P*i* and SD*j*, that if there are more SAPs, then more authentication keys (PS*ij*) should be derived by the P*i* and SD*j*.

Figure 6, Figure 7 and Figure 8 show that applying various distributions (gamma, hypo-exponential and exponential) as residence times did not change the trend of Ψ (*n*, *m*), *E*(*N*) or *C*(*m*) significantly. Therefore, studying the extent of the influence of one of these probability distributions was sufficient. Where the exponential distribution was good in the mean and dealt with all the trends was a special case of the gamma and hypo-exponential distributions.

**Figure 9:**
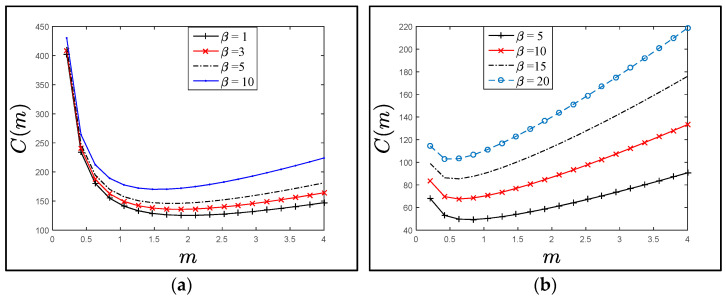

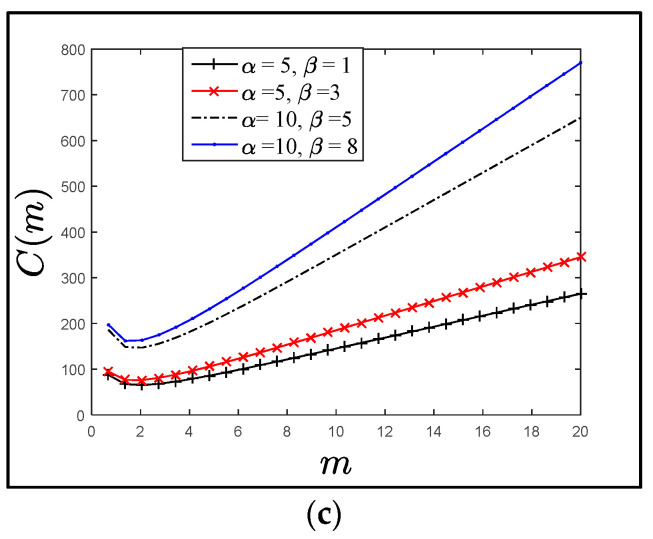
The *C*(*m*) values when the residence time is exponentially distributed with mean *μ*^−1^. (**a**) The *C*(*m*) values when α=10,β≤α. (**b**) The *C*(*m*) values when α=5,β≥α. (**c**) The *C*(*m*) values when α=5,10,β≤α.

Figure 9a–c represent the relation of the *C*(*m*) function when the residence time is exponentially distributed (with mean *µ*^−1^, where *λ* = *µ*) versus *m*-SAP values to illustrate the effect of the overhead transmissions of the authentication messages *α* and *β* during the SAP and WAP execution under different conditions (1 ≤ *β* ≤ *α* =10, *α* = 5 ≤ *β* ≤ 20, and in c, 1 ≤ *β* ≤ 8 and 1 ≤ *α* ≤ 10). All figures show that there is an optimal value X* that minimizes the cost function *C*(*m*), and it increased rapidly after this point. X*=⌈X⌉ can be obtained by differentiating C(*m*) in Equation (8), where X can be approximated by:

(16) $$\gamma^{-X}=1-\left[\frac{5\alpha \left(\ln\gamma \right)}{2\left(\alpha +\beta \right)}\right]+\left(\ln\gamma \right)X$$

## 4. Optimal *m*-Value Selection

This section provides a numerical analysis to compute the optimal values (X*) that minimizes the cost function C(*m*). Applying the Newton–Raphson formula [31] on the derivative of Equation (8), the recursive equation is:

(17) $$X_{k+1}=X_{k}-\frac{2\left(\alpha +\beta \right)+\gamma^{X_{k}}\left[\left(\ln\gamma \right)\left[5\alpha +2\left(\alpha +\beta \right)X_{k}\right]-2\left(\alpha +\beta \right)\right]}{\gamma^{X_{k}}{\left(\ln\gamma \right)}^{2}\left[5\alpha +2\left(\alpha +\beta \right)X_{k}\right]}$$

where X_0_ = 1 and *k* = 0, 1,2, ….

In Table 1, the optimal values X* are given for different *α*, *β*, and *γ*, where *λ* = *zµ*, and *z* = 1, 2, 3, 4, 5, 10, 20, and determined according to different combinations of *α* and *β* values. We assumed that the values of (*β*) were {1, 2, 3, 4, 5, 10, 15, 20, 75, 100} and the values of *α* were {1, 5, 10, 20, 100}. Clearly, the value of X* increased when the ratio (*γ*) increased (i.e., *λ* increased), and X* increased slightly with the large increase in *α* values for any specific fixed value of the request ratio (*γ*). On the other hand, X* decreased when (*β*) increased. However, the results of Table 1 confirmed the consistency of the relation between the optimal value *C*(*m*), *α*, *β* and *γ* that were previously deduced. In this context, the main factors that increased the authentication requests were the medical status and the number of the patient’s connected sensors.

## 5. Conclusions

In the E2EA scheme, it is important to determine an appropriate *m* value that represent how many times the SAPs and WAPs will be executed when the LAP is executed. This can maintain a specific level of security and reduce the authentication signaling traffic cost. In this paper, we proposed an analytical model based on the Poisson process for E2EA to derive the authentication cost function and compute the optimal values of *m* according to the overhead transmission of authentication messages that minimize the signaling traffic cost. We observed from the numerical analysis of the proposed model that the optimal value *m* increased when the value of the authentication request ratio *γ* increased. For any specific *γ* value, the optimal *m* value decreased when the overhead of the authentication messages α transmitted through the communication channels increased. Hence, the service provider of the E2EA scheme-based healthcare IoT system should use an *m*-selection algorithm to determine its optimal value dynamically according to the authentication request ratio of the physician when it executes the LAP and SAP for a specific patient to reduce the cost of authentication signaling traffic. Therefore, investigating of our analytical model using a complement simulation tool, and designing a dynamic algorithm to determine the optimal values of (*m*) with variant authentication request ratio are our future works.

## Figures and Tables

**Table 1:** The optimized X* of the cost function C(*m*) for different values of (*α*) and (*β*) with respect to a fixed ratio (*γ*) when *λ* = *zµ*, where *z* = 1, 2, 3, 4, 5, 10, and 20.

	λ=	μ	2μ	3μ	4μ	5μ	10μ	20μ
α	β	γ=0.5	γ=0.667	γ=0.75	γ=0.8	γ=0.833	γ=0.909	γ=0.952
1	1	2	3	3	3	4	5	7
	2	2	2	3	3	3	4	6
	3	2	2	2	3	3	4	5
	4	2	2	2	2	3	4	5
	5	1	2	2	2	3	3	4
5	1	2	3	4	4	5	6	9
	3	2	3	3	4	4	6	8
	5	2	3	3	3	4	5	7
10	1	2	3	4	4	5	7	9
	2	2	3	4	4	5	6	9
	5	2	3	3	4	4	6	8
	8	2	3	3	4	4	5	8
	10	2	3	3	3	4	5	7
20	1	3	3	4	4	5	7	10
	2	2	3	4	4	5	7	9
	5	2	3	4	4	5	6	9
	10	2	3	3	4	4	6	8
	15	2	3	3	4	4	6	8
	20	2	3	3	3	4	5	7
100	1	3	3	4	5	5	7	10
	10	2	3	4	4	5	7	9
	15	2	3	4	4	5	7	9
	50	2	3	3	4	4	6	8
	75	2	3	3	4	4	6	8
	100	2	3	3	3	4	5	7

## Data Availability

Not applicable.
